# Unusual Coexistence of Takayasu Arteritis, Diffuse Coronary Aneurysms, and a Large Left Atrial Myxoma in an Elderly Male: A Case Report and Literature Review

**DOI:** 10.7759/cureus.110903

**Published:** 2026-06-15

**Authors:** Aoumar G Chamma, Wendy Saliba, Linda Chamma

**Affiliations:** 1 Cardiology, University of Balamand, Beirut, LBN; 2 Urology, Lebanese University, Beirut, LBN

**Keywords:** cardiac mass tumor, coronary artery aneurysms, large atrial myxoma, left atrial mass, takayasu arteritis (ta)

## Abstract

Takayasu arteritis (TA) is a form of vasculitis that primarily affects the large arteries of the body. Involvement of the coronary arteries is uncommon, whereas cardiac myxomas are benign growths within the heart that can cause problems by embolization or by obstruction. Both diseases are rare, and coexistence of both is exceptionally uncommon.

An 84-year-old man with a 30-year history of TA (type V) on low-dose prednisone and weekly methotrexate was found to have a large left atrial myxoma in addition to diffuse coronary aneurysms on routine follow-up. The increased inflammatory markers, erythrocyte sedimentation rate (ESR) 52 mm/h and C-reactive protein (CRP) 18 mg/L, and magnetic resonance imaging (MRI) of the thoracic aorta (wall thickening with mural enhancement of the ascending aorta and arch) confirmed active vasculitis. His modified National Institutes of Health (NIH) score was 5 (active TA). Echocardiography revealed a mass, measuring 3.5 × 3.0 cm in size, arising from the left atrium.

The mass caused mild mitral inflow obstruction resulting in a mean transmitral gradient of 3.5 mmHg across the valve and mild to moderate regurgitation. The estimated pulmonary artery pressure was mildly elevated at 34 mmHg. The patient was, however, asymptomatic and had a good functional status. The coronary arteries were significantly dilated. The left anterior descending (LAD) artery was chronically occluded, and the right coronary artery (RCA) was dilated, forming a huge aneurysm. Given the patient’s age, any surgical approach would be associated with prohibitive risk. Thus, management of the coronary artery abnormalities in this patient with active TA consisted of immunosuppressive therapy, anticoagulation, and guideline-directed medical therapy (GDMT) for hyperlipidemia.

This case highlights the importance of a comprehensive inflammatory and hemodynamic assessment of the patient with TA before any surgical intervention can be considered. In an elderly patient with active disease and in stable hemodynamic condition, even a large, mobile atrial myxoma with mild hemodynamic impact may be managed conservatively with anticoagulation and close surveillance when surgical risk is prohibitive.

## Introduction

Takayasu arteritis (TA) and cardiac myxoma are both exceptionally rare conditions, making their coexistence in one patient unusual. Each has an annual incidence on the order of only 0.5-2.6 cases per million people [1,2]. The chronic large-vessel vasculitis, also known as TA, primarily affects young women who develop symptoms at an average age of 30. The disease affects fewer than 15% of patients, who develop symptoms after age 40 [1]. Cardiac myxoma is a rare tumor in the general population with an incidence of 0.5-1 case per million people per year [2].

Both diseases are serious and may be associated with severe and even life-threatening complications, including stenosis, occlusion, or aneurysm of major arteries in TA, and embolism or obstruction of intracardiac flow in cardiac myxoma. However, both diseases are rare, and no connection between TA and cardiac myxoma has been established. Thus, the coincidence of TA and a cardiac myxoma most likely is a chance event [3].

Coexistence of TA and a cardiac myxoma is rare and is a challenging situation for management. An active state of vasculitis increases the risk of severe and unusual complications in a surgical setting. Patients are highly sensitive to general anesthesia due to systemic inflammation. Also, they are at high risk of anastomotic complications or poor wound healing because of fragile vasculature. It is crucial to confirm the disease activity by clinical parameters, laboratory markers including erythrocyte sedimentation rate (ESR) and C-reactive protein (CRP), and imaging studies, including magnetic resonance imaging (MRI), before any cardiac intervention is planned.

We report an 84-year-old male with longstanding TA who was found to have diffuse coronary artery aneurysms and a large left atrial myxoma. This case underlines the importance of individualized management in a patient with multiple rare co-morbidities, and we review the relevant literature on coronary involvement in TA and the management of cardiac myxomas in high-risk patients.

## Case presentation

An 84-year-old man of Middle Eastern ethnicity presented with a 30-year history of TA (type V vasculitis). He was on a low dose of prednisone (5 mg/day) and methotrexate (15 mg/week). He was a non-smoker with well-controlled hypertension and had no history of coronary artery disease, stroke, or any previous cardiac interventions. He was fully active with no shortness of breath, angina, or syncope in his daily activities. His pulses in the upper and lower extremities were asymmetric. The right brachial pulse was weak compared with the left. There were no cardiac murmurs other than a very soft mid-diastolic murmur heard at the apex of the heart, consistent with mild mitral inflow obstruction.

Initial laboratory testing showed elevated inflammatory markers, including an ESR of 52 mm/h and a CRP level of 18 mg/L (Table 1).

**Table 1:** Laboratory findings at presentation.

Laboratory Parameter	Patient Value	Reference Range
Erythrocyte sedimentation rate (ESR)	52 mm/h	0-20 mm/h
C-reactive protein (CRP)	18 mg/L	<5 mg/L

MRI of the thoracic aorta showed that the ascending aorta and arch had concentric wall thickening and mural enhancement, which confirmed that his TA was active. Based on the modified National Institutes of Health (NIH) criteria, including fever, elevated ESR and CRP, vascular imaging abnormalities, and constitutional symptoms, the disease was categorized as active (score = 5).

As part of surveillance for his cardiovascular status, a transthoracic echocardiogram (TTE) was performed. His TTE revealed a large, mobile mass in the left atrium measuring approximately 3.0 × 3.5 cm (Figure 1).

**Figure 1:**
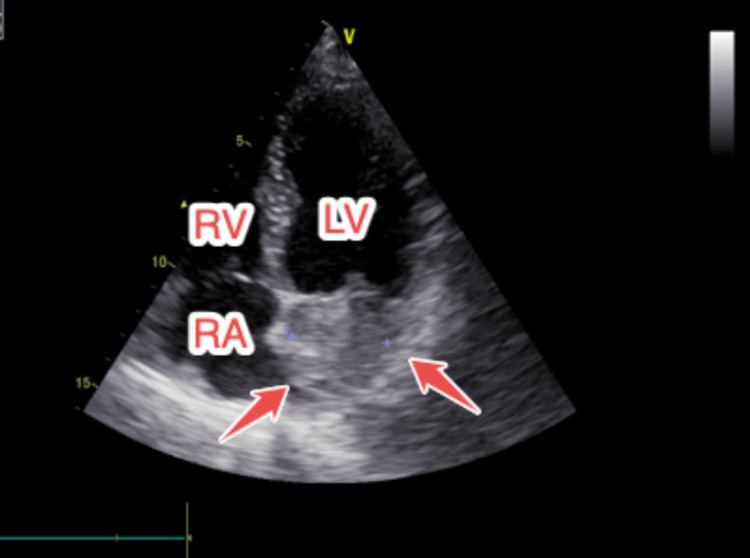
Transthoracic echocardiogram in four chamber view showing a large, left atrial mass measuring approximately 3.0 × 3.5 cm. RA: right atrium; RV: right ventricle; LV: left ventricle

A transesophageal echocardiogram (TEE) was subsequently performed to confirm these findings, providing clearer visualization of the tumor’s attachment and mobility (Figure 2).

**Figure 2:**
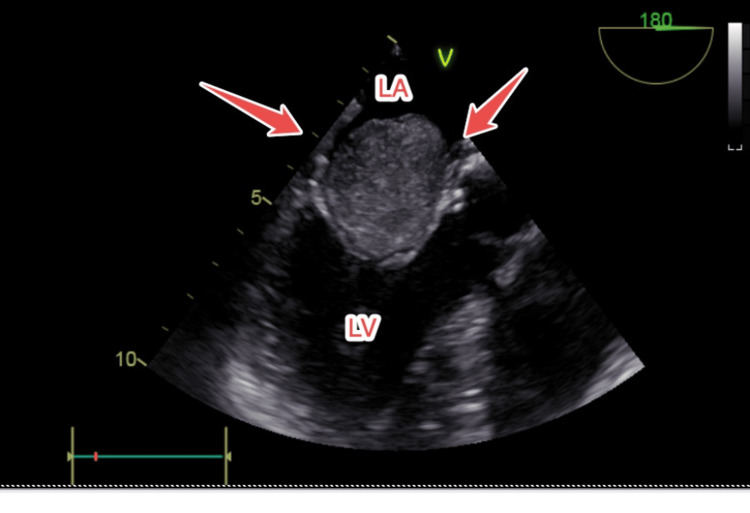
Transesophageal echocardiography demonstrating a mobile cardiac tumor, highlighted by arrows, and its site of attachment. LA: left atrium; LV: left ventricle

On TEE, the tumor stalk was identified as originating from the fossa ovalis region of the interatrial septum, which is the most common site of attachment for left atrial myxomas. Doppler assessment revealed mild-to-moderate mitral regurgitation and a mean transmitral gradient of 3.5 mmHg, with an estimated pulmonary artery systolic pressure of 34 mmHg. There was resultant mild-to-moderate mitral regurgitation and marked left atrial enlargement (Figure 3).

**Figure 3:**
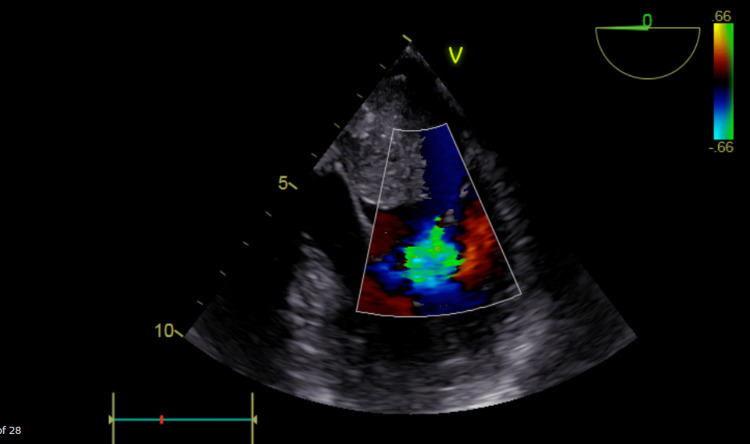
Transesophageal echocardiography showing mild-to-moderate mitral regurgitation and marked left atrial enlargement.

Left ventricular systolic function was reduced (ejection fraction (EF) around 50%) with regional wall-motion abnormalities (apical hypokinesia).

Given the low mean transmitral gradient of 3.5 mmHg, the absence of positional symptoms, and the need to evaluate functional ischemia before committing to a management strategy, a supervised treadmill stress test was deemed acceptable, with the team acknowledging the theoretical risk of hemodynamic compromise from tumor mobility during exercise. The patient exercised for 10 minutes on the Bruce protocol and stopped because of fatigue. He remained asymptomatic. The resting electrocardiogram demonstrated normal sinus rhythm (Figure 4).

**Figure 4:**
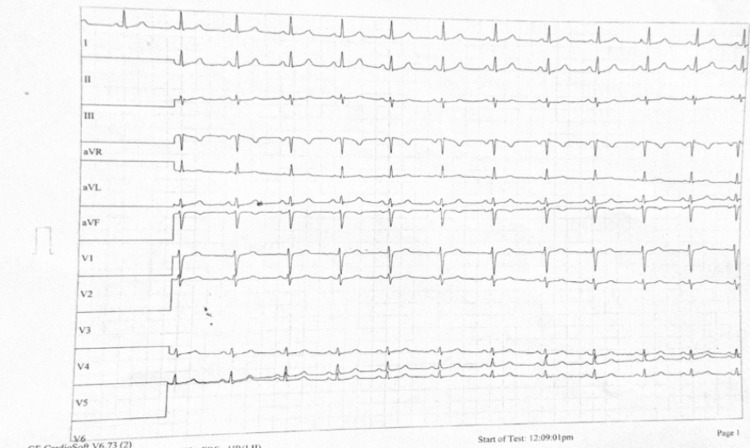
Resting electrocardiogram showing normal sinus rhythm prior to exercise stress testing.

After 10 minutes of exercise, he developed a new left bundle branch block (LBBB) that persisted at peak exercise and into early recovery (Figure 5). Given the new stress-induced LBBB, invasive coronary angiography was pursued.

**Figure 5:**
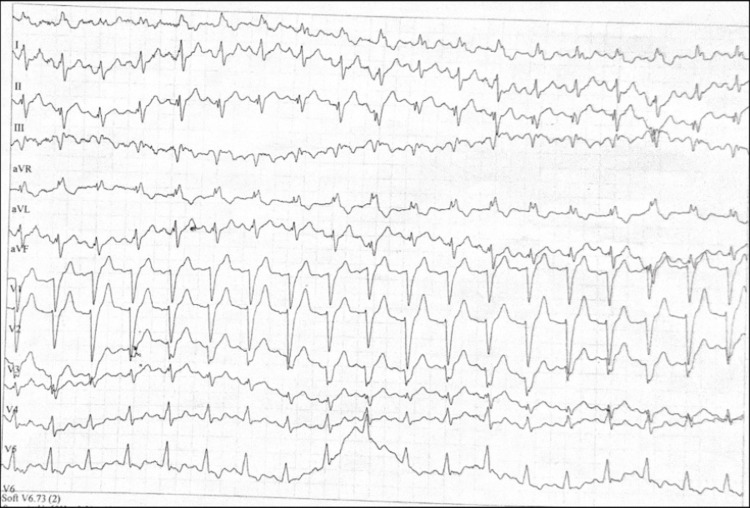
Electrocardiogram at peak exercise demonstrating new-onset left bundle branch block.

Coronary angiography showed diffuse aneurysmal disease involving the left coronary system (Figure 6) and the right coronary artery (RCA) (Figure 7).

**Figure 6:**
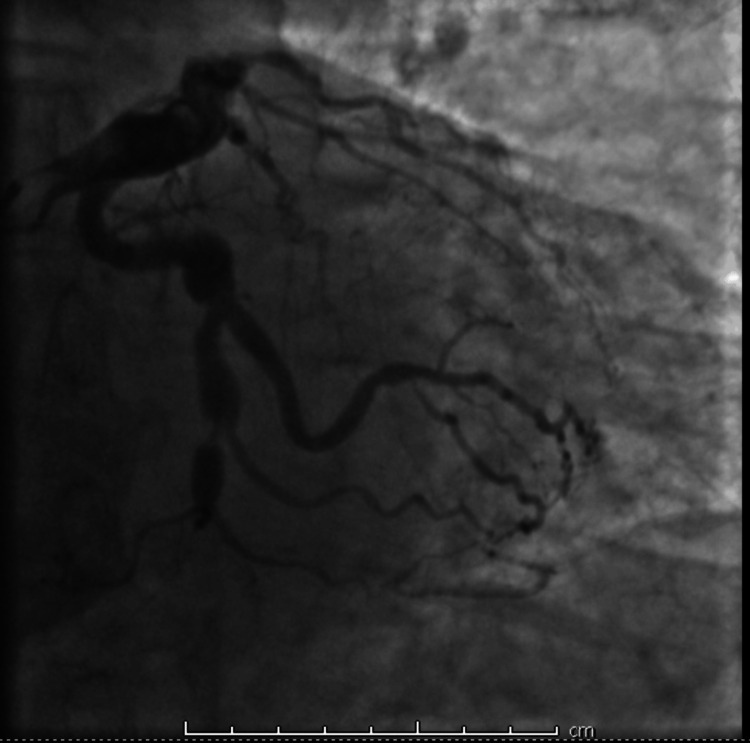
Coronary angiography of the left coronary system showing diffuse aneurysmal disease involving all major coronary arteries.

**Figure 7:**
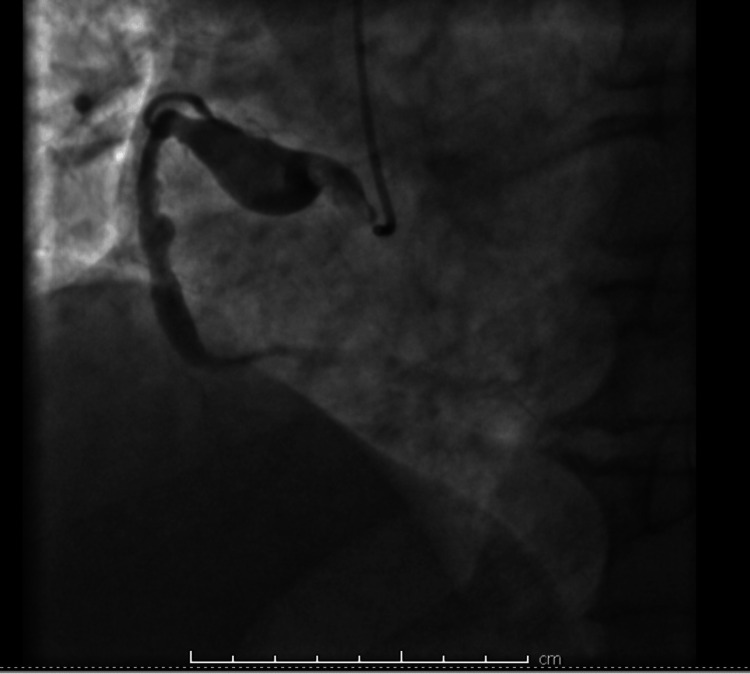
Coronary angiography of the right coronary artery showing diffuse aneurysmal disease involving the entire right coronary artery course.

The mid-left anterior descending artery (LAD) was chronically occluded (Figure 8).

**Figure 8:**
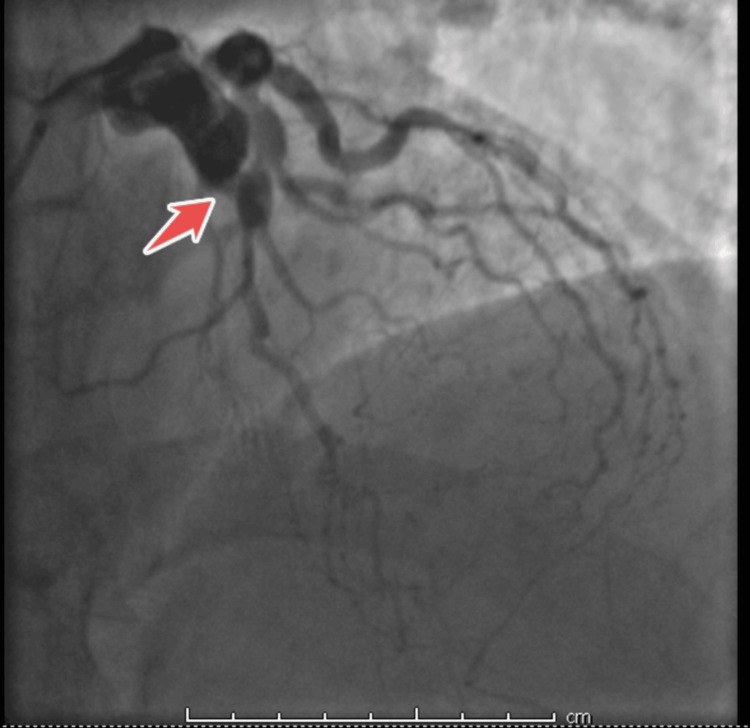
Coronary angiography of the left anterior descending artery showing diffuse aneurysmal disease with chronic total occlusion (arrow).

The overall coronary anatomy was markedly abnormal and high-risk for revascularization. These findings suggested that the left ventricular dysfunction likely reflected chronic ischemic injury related to coronary involvement from TA. There were no active thrombotic lesions, and collateral circulation appeared well developed.

The case was discussed in a multidisciplinary team including cardiology, cardiothoracic surgery, and rheumatology. The decision was challenging because the patient presented with two critical cardiac problems consisting of a large atrial myxoma and widespread coronary artery disease while suffering from active large-vessel vasculitis (TA) as an elderly, frail patient. Surgical excision of the myxoma (via open-heart surgery) combined with any form of coronary revascularization (bypass grafting) would be extremely high-risk given the patient’s advanced age and the complex coronary anatomy (aneurysmal, fragile vessels). Even isolated tumor resection posed substantial risk due to the need for cardiopulmonary bypass and manipulation of a heart with diffusely diseased coronaries. Furthermore, TA can complicate surgical outcomes; active inflammation in large vessels might impair healing or precipitate complications, and prior studies have noted poor results with surgical interventions during active disease. Considering these factors, the consensus was that the risks of immediate surgery outweighed the benefits in this particular case.

Given that the patient was asymptomatic from the atrial myxoma (no evidence of tumor-related embolization or obstruction aside from mild mitral regurgitation) and had stable heart failure without angina, the decision was made to pursue a conservative medical management strategy. The myxoma would be managed non-operatively with close surveillance, and the coronary artery disease and heart failure would be treated medically. The patient and family were in agreement with this cautious approach.

Accordingly, the patient was started on comprehensive guideline-directed medical therapy for heart failure with reduced EF and secondary prevention of cardiac events. This included an angiotensin-converting enzyme (ACE) inhibitor, a beta-blocker, a mineralocorticoid receptor antagonist, and a sodium-glucose cotransporter 2 (SGLT2) inhibitor. Also, the prednisone dose was increased to 10 mg daily due to active TA, with the continuation of methotrexate weekly with the same dose. In addition, low-dose aspirin and a high-intensity statin were initiated for secondary prevention given his diffuse coronary disease (recognizing that even non-atherosclerotic aneurysmal vessels benefit from anti-thrombotic and plaque-stabilizing therapy). Because of the large left atrial mass and dilated left atrium (which confer risk of clot formation and systemic emboli), anticoagulation with apixaban was started to reduce the risk of tumor-related thromboembolism. His chronic immunosuppressive therapy for TA (low-dose prednisone plus methotrexate) was continued to maintain disease remission.

The patient was discharged home in stable condition with careful follow-up arrangements. At three-month review, he reported improved exercise tolerance (mild fatigue but no exertional chest pain or syncope). Repeat echocardiography at that time showed findings similar to prior: the atrial myxoma was unchanged in size and mobility, mitral gradient had not changed, pulmonary artery pressure (PAP) had decreased to 28 mmHg, and left ventricular function remained around 50% EF without new wall-motion abnormalities. There were no signs of tumor embolization or progression of valvular obstruction. Given the favorable short-term outcome, plans were made for serial echocardiographic monitoring every three to six months. The team recommended surgical intervention only when the myxoma showed substantial growth or when it led to heart failure or valve blockage or when it caused blood clots to form. The goal was to optimize medical treatment while reducing the need for invasive procedures in this patient who had multiple high-risk factors.

## Discussion

TA is a form of immune-mediated granulomatous vasculitis that almost exclusively affects the aorta and its major branches. It can cause stenosis or aneurysm of involved vessels due to inflammation of the arterial wall and subsequent fibrosis. Coronary artery involvement in TA is not common and has been reported in up to 10% of cases in several retrospective studies [4]. Most of the reported cases of coronary artery lesions are of ostial lesions, followed by diffuse type of narrowing or arteritic occlusion of the coronary arteries. Coronary artery aneurysms are extremely rare and have been reported in fewer than 1.5% of cases of TA [4]. Our patient has diffuse aneurysmal dilatation of all major coronary arteries with a giant aneurysm of the RCA and occlusion of the LAD artery. This is an unusual presentation of coronary involvement in TA. Similar cases have been reported only infrequently. For example, Ouali et al. described a 19-year-old man with TA who presented with an acute myocardial infarction due to a giant aneurysm of the RCA causing occlusion; that patient had multiple other aneurysms (carotid, vertebral, renal arteries) in the setting of active TA [5]. Such combinations of coronary aneurysm and occlusion in Takayasu are uncommon.

The pathogenesis of coronary artery aneurysm in TA can be multifactorial [4]. Chronic inflammation of the aortic wall can extend into the proximal segments of the coronaries, which leads to its weakening, followed by aneurysmal dilation [4]. Some authors have also suggested that systemic inflammation and hypertension that persists for long periods in TA patients may lead to faster atherosclerotic progression, which results in coronary artery ectasia and aneurysm development [4]. In general populations, coronary aneurysms are most often observed in the RCA due to atherosclerosis, but interestingly, in TA, both left and right coronaries appear equally likely to be affected by aneurysmal changes [4]. The presence of a coronary aneurysm is clinically significant; these enlarged blood vessels form thrombi that cause distal embolism or vessel rupture, which increase mortality rates in these patients [4]. The diagnosis of coronary aneurysms requires healthcare providers to evaluate other possible causes of the condition. Kawasaki disease in children, severe atherosclerosis, and polyarteritis nodosa are well-known causes of coronary artery aneurysm or ectasia [4].

Management of coronary involvement in TA is individualized and decided on a case-by-case basis. In the minority of cases where there is critical coronary ostial stenosis presenting with refractory angina or myocardial infarction, management may involve an attempt at revascularization, either by surgical bypass or percutaneous coronary angioplasty, in conjunction with treatment of the active vasculitis [4]. However, diffuse aneurysmal disease without focal stenosis poses a therapeutic challenge. Stenting or bypass grafting aneurysmal, non-stenotic segments is usually not beneficial and can be high risk [4]. In patients with active TA, surgery involving affected blood vessels is technically challenging and may result in graft failure or complications at the anastomotic sites [4]. Our patient was asymptomatic and therefore managed conservatively with heart failure therapy and close clinical follow-up. Any percutaneous or surgical intervention on his coronary arteries would have carried a high risk and was therefore not indicated. As with most patients with TA, the cornerstone of management remains medical therapy and close clinical surveillance.

If at any time the patient develops angina, progressive aneurysmal enlargement of his coronary arteries, or deterioration in his coronary status, or if the left atrial myxoma becomes hemodynamically significant, the need for intervention would need to be reassessed.

Cardiac myxomas, found in the left atrium, are considered the most benign cardiac tumor [6]. It can be associated with embolic phenomena due to a stroke or systemic emboli from the tumor fragments; also, sometimes these myxomas can cause mitral valve inflow obstruction leading to syncope or even heart failure, and many other constitutional symptoms like fever and weight loss, due to interleukin-6 production by the tumor [6]. In our case, it was an incidental finding, and there were no major symptoms. However, there was a risk for serious complications due to its large size (3 × 3.5 cm) and mobility. Standard management of atrial myxoma is surgical resection, even if the patient is asymptomatic [6]. Surgical removal is considered curative and prevents the dreaded outcomes of stroke or sudden cardiac arrest from acute obstruction [7]. In fact, conventional teaching has regarded atrial myxoma as a surgical emergency once diagnosed; expeditious removal is recommended because of the risk of embolism at any time, but in practice, the urgency of surgery may be tailored to the patient’s condition [7]. Pinede et al. reported that about half of myxoma cases present in a stable condition without immediate life-threatening features [8]. More recently, Selkane et al. observed that with advances in echocardiography, myxomas are increasingly identified at earlier stages in older patients, and the classic approach of emergent surgery may not always be necessary for stable tumors [9].

Our patient’s situation represented a rare instance where surgical resection was deemed too high-risk, forcing us into a non-standard conservative management plan. The decision was influenced by multiple factors like his advanced age and frailty, his underlying vasculitis, and the presence of diffuse coronary aneurysmal disease, making open-heart surgery and grafting perilous. Importantly, his tumor had not yet caused any embolic or obstructive complications, and we planned vigilant surveillance. We balanced the immediate risk of surgery against the risk of observation. The risk of not removing a large myxoma is primarily the chance of embolization [10,11]. To mitigate embolic risk, we instituted anticoagulation, though it’s unproven whether anticoagulation definitively prevents tumor fragment emboli; it can prevent thrombus formation on the tumor surface [10,11].

Our plan relies on frequent imaging follow-up. We have arranged echocardiograms every three to six months to watch for any growth of the myxoma or new impact on mitral valve function. If at any point the tumor increases in size or mobility, or if the patient develops even a minor embolic event, the calculus would change. This case also highlights the importance of a multidisciplinary approach, because involving all relevant specialties ensures that the treatment plan is well-rounded and all contingencies are considered.

## Conclusions

This case illustrates an exceedingly unusual coexistence of three pathologies - TA, diffuse coronary artery aneurysms, and a large left atrial myxoma - in an elderly male patient. Each condition on its own is rare, and to our knowledge, their combination in one individual has not been previously reported in the literature. The management of such a patient must be highly individualized. In our case, the patient’s advanced age and comorbid diffuse coronary vasculopathy rendered the standard surgical treatment of the cardiac myxoma prohibitively risky. Instead, we went for a medical conservative approach. This strategy, while deviating from the standard approach, was guided by multidisciplinary consensus. Early follow-up has been encouraging, with the patient remaining stable and the tumor unchanged on serial imaging.

This case highlights several important lessons. For example, when patients with TA develop unexplained heart failure or ischemia, dedicated coronary imaging should be considered to reveal possible aneurysms. Usually, immediate surgery is indicated in patients with myxoma, but a nuanced approach should be considered if these patients are at high-risk; and vigilant surveillance with adjunctive medical therapy may be considered then. Our patient will continue to be clinically observed, and the decision of surgical intervention remains on the table if his myxoma enlarges or becomes symptomatic. This case underscores that in the era of modern imaging and therapies, even “surgical” diseases like atrial myxoma can occasionally be managed conservatively when patient factors demand it, though such decisions must be made cautiously and on a case-by-case basis.

## References

[1] Roberts JR, Monteagudo LA (2024). Takayasu arteritis: practice essentials, background, pathophysiology. Medscape.

[2] Ashinze P, Banerjee S, Egbunu E (2024). Cardiac myxomas: a review of current treatment approaches and emerging molecular therapies. Cardiothorac Surg.

[3] Tan CY, Qin W, Lin H, Wang ZM, Xie QB, Liu Y (2010). Atrial myxoma with metastasis misdiagnosed as Takayasu arteritis. Rheumatol Int.

[4] Ayyappan A, Gopalakrishnan A, Pitchai S (2021). Concurrent coronary artery and subclavian arterial aneurysms in Takayasu arteritis. Egypt J Radiol Nucl Med.

[5] Ouali S, Kacem S, Ben Fradj F (2011). Takayasu arteritis with coronary aneurysms causing acute myocardial infarction in a young man. Tex Heart Inst J.

[6] Karabag T, M Dogan S, Aydin M, R Sayin M (2011). Giant left atrial myxoma causing mitral valve obstruction and concomitant coronary artery disease. Cardiol Res.

[7] Sharma GK (2022). Atrial myxoma treatment & management: approach considerations, medical care, surgical care. Medscape.

[8] Pinede L, Duhaut P, Loire R (2001). Clinical presentation of left atrial cardiac myxoma. A series of 112 consecutive cases. Medicine (Baltimore).

[9] Selkane C, Amahzoune B, Chavanis N, Raisky O, Robin J, Ninet J, Obadia JF (2003). Changing management of cardiac myxoma based on a series of 40 cases with long-term follow-up. Ann Thorac Surg.

[10] Chamma AG, Saliba W, Chamma L (2025). Listeria monocytogenes infective endocarditis with concurrent bacterial meningitis and systemic embolization: a case report and literature review. Cureus.

[11] Chen Y, Huang Q, Bai C, Zhang H, Zhang B (2024). Preoperative management and anticoagulant efficacy in atrial myxoma-associated acute ischemic stroke: a case report and literature review. Front Cardiovasc Med.

